# Natural zwitterionic betaine enables cells to survive ultrarapid cryopreservation

**DOI:** 10.1038/srep37458

**Published:** 2016-11-22

**Authors:** Jing Yang, Nana Cai, Hongwen Zhai, Jiamin Zhang, Yingnan Zhu, Lei Zhang

**Affiliations:** 1 Department of Biochemical Engineering, School of Chemical Engineering and Technology, Tianjin University, Tianjin 300072, P. R. China; 2 Key Laboratory of Systems Bioengineering of the Ministry of Education, Tianjin University, Tianjin 300072, P. R. China; 3 Collaborative Innovation Center of Chemical Science and Engineering (Tianjin), Tianjin University, Tianjin, 300072, P. R. China

## Abstract

Cryoprotectants (CPAs) play a critical role in cryopreservation because they can resist the cell damage caused by the freezing process. Current state-of-the-art CPAs are mainly based on an organic solvent dimethyl sulfoxide (DMSO), and several DMSO-cryopreserved cell products have been brought to market. However, the intrinsic toxicity and complex freezing protocol of DMSO still remain as the bottleneck of the wide use for clinical applications. Herein, we reported that betaine, a natural zwitterionic molecule, could serve as a nontoxic and high efficient CPA. At optimum concentration of betaine, different cell types exhibited exceptional post-thaw survival efficiency with ultrarapid freezing protocol, which was straightforward, cost efficient but difficult to succeed using DMSO. Moreover, betaine showed negligible cytotoxicity even after long-term exposure of cells. Mechanistically, we hypothesized that betaine could be ultra-rapidly taken up by cells for intracellular protection during the freezing process. This technology unlocks the possibility of alternating the traditional toxic CPAs and is applicable to a variety of clinical applications.

Cryopreservation is the most reliable method for the long-term storage of biological samples, such as cells, tissues and organs, etc.^1^,^2^,^3^. At cryogenic temperature, bio-samples enter a ‘suspended animation’ state, and their ‘life clock’ is halted in cryopreservation for a couple of days, several years, or even decades, until they are recovered after a proper thawing procedure to physiological temperature^4^,^5^. Currently, cryopreservation technology is an escalating need for increasing clinical demands of organ transplantation and regenerative medicine. For example, by the end of year 2008, it had exceeded 100 thousands candidates on the organ waiting list with only less than 30 thousands donors in the United States, so preservation failure of organs is highly unfavorable^6^,^7^. Moreover, with more than 600 thousands cell therapeutic units manufactured and 30 thousands patients treated, the efficient cryopreservation is responsible for the successful organ transplantation and improved utilization of cell therapies^8^,^9^. It is also expected that in the future, cryopreservation may also offer the possibility for long-term preservation of whole human bodies in order to realize ‘resurrection’ or ‘interstellar travels’, etc.^10^,^11^.

Besides pausing the biological activities, the freezing process to cryogenic temperature can inevitably induce ice formation, which can irreversibly cause mechanical damage (known as ice injury) and osmotic shock (known as solute injury) to the cells (Fig. 1A)^12^. To solve this problem, cryoprotectants (CPAs) have been developed to minimize or even eliminate ice formation, thus they are now essential for successful cryopreservation^13^. Dimethyl sulfoxide (DMSO) (Fig. 1B) is the most widely used CPA for the cryopreservation of various biosamples due to its high cryoprotect efficacy^14^. Moreover, several DMSO-cryopreserved therapeutic cell products are already commercially available (e.g. adult human mesenchymal stem cells, Osiris Therapeutics). However, DMSO has been found to present several fatal drawbacks greatly hampering its clinical applications. Firstly, DMSO is also a commonly used organic solvent with intrinsic toxicity. It can induce the cell apoptosis even at a very low concentration, and can also lead to uncontrolled differentiation of stem cells^15^. In recent clinical studies, DMSO is considered as the main culprit in a variety of adverse events on patients for stem cell transplantations, such as allergys, gastrointestinal complaints, neurological complaints, and renal dysfunctions, etc.^16^. Even more concerning, after infusion of DMSO-cryopreserved hematopoietic stem cells, 97 out of 144 patients (67.36%) were found to develop adverse events^17^. Secondly, traditional DMSO-cryopreserved protocol is time consuming and complex. Stepwise freezing protocols (normally take more than 12 hours) are essential for DMSO transmembrane diffusion into the cells to prevent intracellular ice formation and achieve optimized intracellular bioprotection^18^. Although ultrarapid freezing protocol (only take a few minutes or less) is a much more efficient and favorable option, this is still a big challenge for current CPAs including DMSO^19^,^20^,^21^,^22^,^23^,^24^. Thirdly, cells are highly vulnerable to the osmotic shock during the introduction and removal of large amount of DMSO (commonly 10–15%) in freezing and thawing processes^25^.

Ideal CPAs should be more efficient while avoid the detrimental effects. Actually, nature does offer us several important inspirations though no significant progress has been made. Antifreeze proteins (AFPs) found in Antarctic fishes are one most representative example, but it is reported that AFPs are immunogenic, difficult to prepare, and most importantly, for cryopreservation they offer limited benefit^26^,^27^,^28^,^29^. Synthetic polymers were tested as AFP mimics, but only ~40% post-freezing recovery rate can be achieved for erythrocyte cryopreservation^30^. Another example is trehalose (Fig. S1A), a natural nonreducing disaccharide of glucose (Fig. S1B) that has been applied to the bioprotection of a variety of biologicals, including proteins, vaccines, and bacteria, etc.^31^,^32^. However, trehalose alone cannot work well in cryopreservation due to its poor permeability into the cells. Several reports have shown that its cryoprotect efficacy is improved if it can be delivered into the cells with various strategies, such as microinjection, pore formation using mutant bacterial toxins, or internal trehalose synthesis via genic engineering, etc.^33^,^34^,^35^.

Inspired by nature, we found a natural molecule—betaine can work as a highly promising CPA. Betaine is a zwitterionic and hydrophilic molecule (Fig. 1C); it is rich in abundant microorganisms, plants, and animals. In human serum, the resting concentration of betaine ranges from ~2.3 to 8.2 mg/L^36^. As a well-known osmoprotectant, betaine is able to protect cells against osmotically induced inactivation^37^. For example, during salmon juveniles migrate from freshwater hatcheries to the high salinity of seawater, large amount uptake of betaine can moderate the osmotic pressure change to significantly improve their viability^38^. Moreover, it has been reported that zwitterionic betaine could form a monolayer of water around the proteins to maintain their stability and functions^36^. Most interestingly, betaine is also associated with the freezing tolerance of various plants. Kishitani *et al*. found that the winter-type barley could accumulate betaine at high levels in their leaves during cold acclimation to protect them from freezing injury^39^. Motivated by these findings, we tested cell cryopreservation using betaine as a nontoxic alternative to DMSO in this work. We also adopted ultrarapid freezing, which was more straightforward than the conventional controlled-rate freezing protocol, to cryopreserve different types of cells, and compared the post-thaw survival efficiency and cytotoxicity to those of DMSO.

## Results and Discussion

### Effect of betaine on ice formation

**Figure 1:**
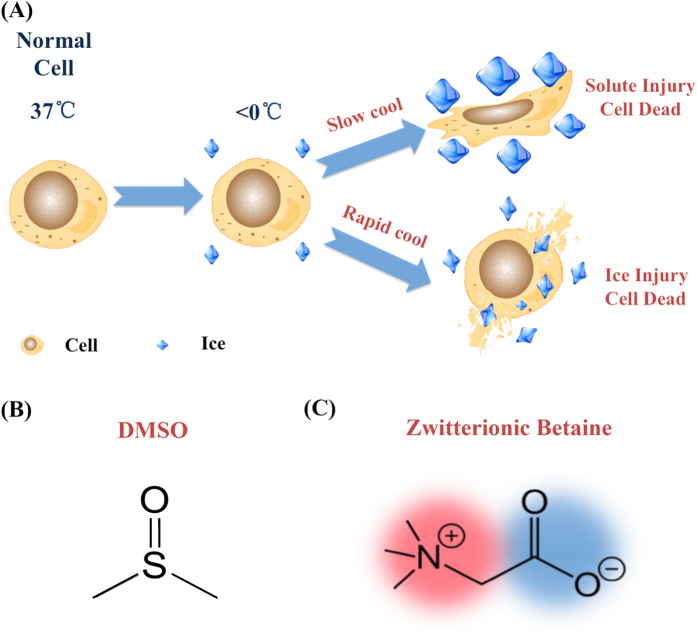
(**A**) Schematic drawing of two types of cryo-injuries in the freezing process. The molecular structure of (**B**) DMSO and (**C**) zwitterionic betaine.

Figure 1A showed the two types of cryo-injuries to cells caused by ice formation: at lower cooling rate, cells are mainly damaged by excessive shrinkage as solute injury, because the extracellular ice growth induce cell exposure to an increasingly hypertonic environment; at higher cooling rate, intracellular ice will quickly form and cells are mainly damaged mechanically by the intracellular ice injury^12^. Therefore, the two most crucial tasks of a CPA are to prevent the solute injury and ice injury during the freezing process.

**Figure 2:**
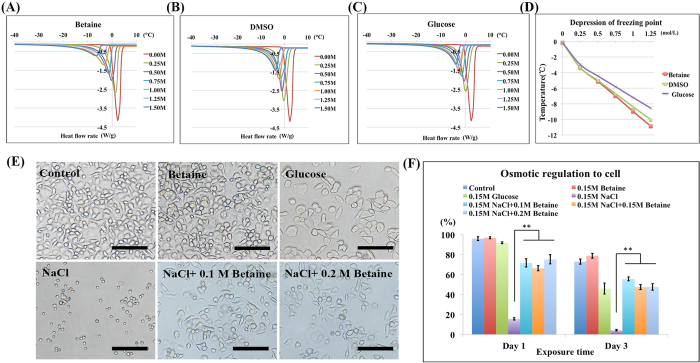
Effects on ice formation and osmotic regulation of betaine. Differential scanning calorimetry (DSC) thermograms of pure water (red), 0.25 M (green), 0.5 M (purple), 0.75 M (light blue), 1 M (orange), 1.25 M (gray), 1.5 M (dark blue) of (**A**) betaine, (**B**) DMSO and (**C**) glucose. (**D**) The depression of water freezing point of betaine (red), DMSO (green) and glucose (purple). (**E**) GLC-82 cell attachment after exposure in medium, 0.15 M betaine, 0.15 M glucose, 0.15 M NaCl, 0.15 M NaCl + 0.1 M betaine, and 0.15 M NaCl + 0.2 M betaine for 3 day. Scale bar = 50 μm. (**F**) The cell viability after exposure in medium (dark blue), 0.15 M betaine (red), 0.15 M glucose (green), 0.15 M NaCl (purple), 0.15 M NaCl + 0.1 M betaine (light blue), 0.15 M NaCl + 0.15 M betaine (orange), and 0.15 M NaCl + 0.2 M betaine (gray) for 1 day and 3 day. Value = mean ± standard deviation, n ≥ 3. p <  *0.05; **0.01; ***0.001.

The interaction of water molecules had shown a precise correlation with ice formation and growth^40^. So to minimize the ice formation and growth, CPAs are commonly hydrophilic molecules that can inhibit water crystallization and decrease the water chemical potential. Zwitterionic betaine is well known for its highly hydrophilic property; it can strongly bind water molecules via ionic solvation effects due to its charged groups^41^. So firstly we studied its influence to ice formation using differential scanning calorimetry (DSC), and compared with DMSO and glucose samples (Fig. 2A–D). All the samples showed an endothermic peak due to the melting of solute-water system, and the peak value was inversely proportional to the concentrations (Fig. 2A–C). Among the three samples, betaine sample showed the smallest melting peak than those of DMSO and glucose at comparable concentrations, indicating its strongest inhibition to water crystallization. Figure 2D revealed the melting point of the three systems at different concentrations, and betaine possessed the strongest ability to depress the water freezing point, suggesting its excellent capability of decreasing the water chemical potential.

### Betaine regulates osmotic stress

Aside from ice injury, solute injury induced by osmotic shock is also responsible for cell death during the freezing and thawing process. To study the osmotic regulation ability of betaine, GLC-82 cells were exposed in hypertonic medium containing betaine, glucose, NaCl, and NaCl/betaine mixtures at physiological temperature for 1 day and 3 days (Fig. 2E,F). When cells were exposed in hypertonic NaCl or glucose medium (both natural products), osmotic shock would induce massive loss of intracellular water (solute injury) and thus lead to cell death (Fig. 2E). Though the cell viability in hypertonic glucose medium was greater than 50% at 3 days, cells presented abnormal morphology as compared with normal cells, as shown in Fig. 2E. In comparison, after cells were exposed in hypertonic betaine medium for 3 days, they could still attach to cell culture substrates and assumed their spindle shape similar with the control cells. Interestingly, adding betaine into the hypertonic NaCl medium was able to significantly improve the viability of cells, without obviously colligative properties of betaine concentrations (Fig. 2F). These phenomena indicated that by accumulating this natural osmoprotectant—betaine, cells would adapt to external osmotic stress. Therefore, betaine was highlighted the uniqueness for protecting cells from solute injury.

### Cell cryopreservation with ultrarapid freezing protocol

In most cases, ultrarapid freezing means plunging samples directly into liquid nitrogen. This technique is simple, highly cost-effective and timesaving compared with conventional slow-freezing techniques^42^. However, current CPAs (e.g. DMSO) often do not work at such high cooling rates, because they require much longer time to enter the cells for intracellular bioprotection. Increasing the CPA concentrations may accelerate cellular permeation, but meanwhile it will inevitably increase the toxic effects^43^.

**Figure 3:**
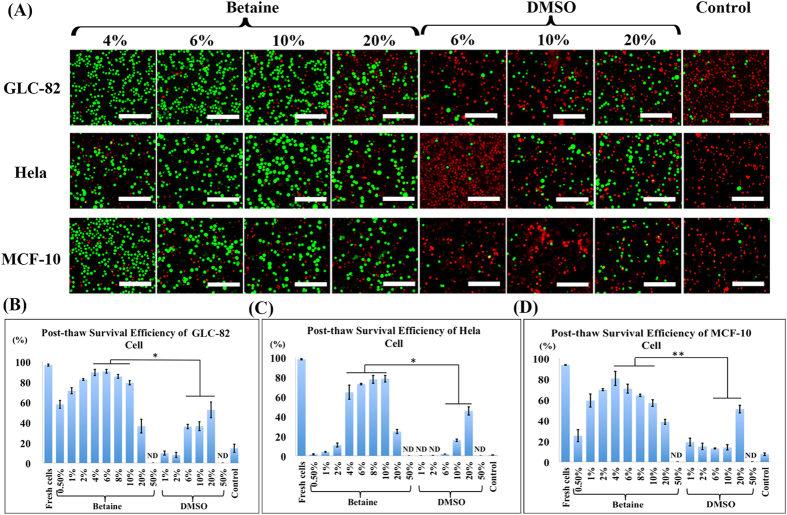
Cell cryopreservation using betaine with ultrarapid freezing. (**A**) Fluorescence images of the live/dead assay of GLC-82 cells (upper row), Hela cells (middle row), and MCF-10 cells (lower row) cryopreservation with different concentrations of CPAs (betaine and DMSO), and cryopreservation in culture medium as control. Post-thaw survival efficiency of (**B**) GLC-82 cells, (**C**) Hela cells, and (**D**) MCF-10 cells evaluated at different concentrations of CPAs and control, with an identical cell amount (1.0 × 10^6^). Green: live cells. Red: dead cells. ND: not detected. Scale bar = 50 μm. Value = mean ± standard deviation, n ≥ 3. p < *0.05; **0.01.

In this work, cryopreservation of three cell types (GLC-82 cells, Hela cells and MCF-10 cells) using betaine was tested with ultrarapid freezing protocol. Fig. 3 presented the post-thaw (37 °C thawing temperature) survival efficiency of the three cell types at different concentrations of betaine or DMSO. A bell-shaped relationship could be observed between betaine concentration and cell survival efficiency. As shown in Fig. 3B, the optimum efficiency of GLC-82 cells was 90.4% found at 6% of betaine. While DMSO, the most commonly used CPA, showed a significantly lower efficiency of GLC-82 cells than betaine at the comparable concentrations. It could be deduced that in the rapid cooling process, there was not enough time allowing DMSO to permeate into cells for intracellular protection. In addition, at higher DMSO concentration (50%), the increased cytotoxicity will damage the cells even quicker. Meanwhile, the blank control samples containing medium alone showed negligible efficiency. Similarly, the post-thaw survival efficiency of Hela cells and MCF-10 cells were 78.4% and 80.4% at the optimal concentrations of betaine (10% and 4%), respectively, while the efficiency using DMSO and medium alone was significantly lower or even negligible (Fig. 3C,D).

We also evaluated the influence of thawing temperatures. It was found that, as shown in Fig. S2, addition of 4%, 6%, 8%, and 10% betaine with fast thawing temperatures (45 °C water bath) resulted in lower survival efficiency of Hela cells compared with the commonly used thawing temperatures (37 °C water bath), due to the limited toleration of cells in the elevated temperatures. Lowering the thawing rate by decreasing the thawing temperature to 4 °C significantly reduced the survival efficiency, possibly caused by the increased ice recrystallization or ice growth.

We further validated the cell cryopreservation efficiency using betaine at different cell densities. As presented in Fig. S3, addition of 6% betaine to samples with cell density of 1 × 10^6^, 5 × 10^5^, and 1 × 10^5^ cells per 1.5 mL achieved 85.8%, 88.8%, and 90.4% post-thaw cell survival efficiency, respectively. We also explored betaine for the long-term cell cryopreservation (3 months), and results showed that there was no observable difference of the efficiency from 1 day to 3 months (all above 80% at optimum betaine concentrations) (Fig. S4). These results indicated that betaine could work as a versatile CPA for different cell types, cell densities and periods.

### Cytotoxicity evaluation

**Figure 4:**
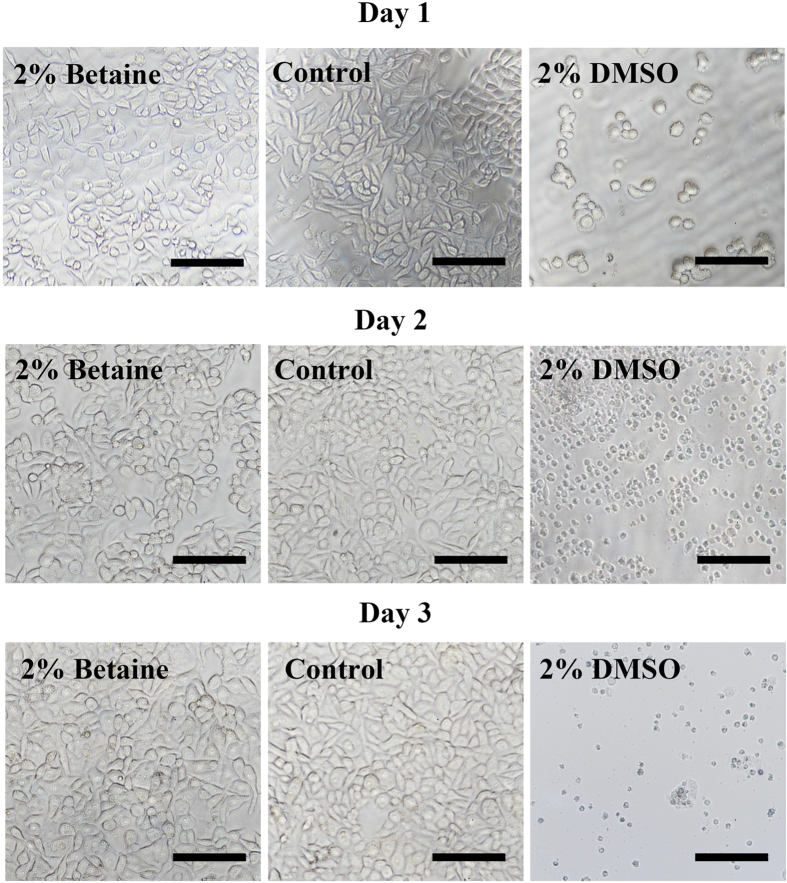
Cytotoxicity tests of betaine and DMSO. The attachment of GLC-82 cells after exposure in medium containing 2% of betaine and DMSO for 1 day (upper row), 2 days (middle row) and 3 days (lower row), and in culture medium as a control. Scale bar = 50 μm.

The toxicity of current CPA stands in the way of various biomedical applications. Major cell damages caused by CPA toxicity are lists as follows: (i) cell membranes are breached or damaged; (ii) enzyme function is impaired; (iii) cell development or proliferation is diminished; (iv) mitochondrial function is reduced; (v) DNA, proteins, or other macromolecules are damaged^44^. These damages will directly induce the dysfunction of cell behaviors, such as attachment or proliferation. Therefore, we respectively exposed GLC-82 cells in medium containing 2% betaine or DMSO at 37 °C from 1 day to 3 days, and then their cell-attachment efficiency was tested. Similar with the control sample in the medium alone, the cells in betaine solutions could attach to cell culture substrates with their normal morphology, indicating the integrity of cell membranes and adhesion proteins (Fig. 4). In comparison, at day 1, only a few cells in 2% DMSO could attach to the substrates while exhibited abnormal morphology; after two days, all the cells were floating with shrinkage appearance, suggesting they were completely dead. This was because DMSO molecules could directly block the actions of membrane channel proteins, and caused plasma membrane blebs that indicated dissociation between the plasma membrane and the cytoskeleton.

It is well known that traditional CPAs must be washed away and replaced with the fresh medium right after recovery to minimize their toxic effects. This step is also essential in current cell therapy, but it notably complicates the therapeutic procedure, hampering the standardization and wide applications of cell therapy. In this work, we were excited to find that the washing step could be skipped when using betaine as the CPA. After cryopreservation, the post-thaw cells with CPA (betaine or DMSO) solution added four-fold amount of medium were directly infused into the culture flask for cell recovery (Fig. S5). Interestingly, without the washing step, the recovery of three cell types with CPA solution (initially containing 6% or 10% of betaine) were all successful, and the recovered cells were found to attach to the substrate with normal cell morphology. Moreover, the recovered cells resumed the proliferation as well as a normal degree of cell division (Fig. S6), also suggesting that basic cell functionalities such as enzymatic or mitochondrial function were not affected. In comparison, the cell suspension with DMSO showed few or even no cell attachment. These results highlighted the unique property of betaine as a nontoxic CPA to achieve standardized therapeutic cell products, which could be infused into patients after thawing without complex washing procedure and potential contaminations.

### Rapid intracellular bioprotection

**Figure 5:**
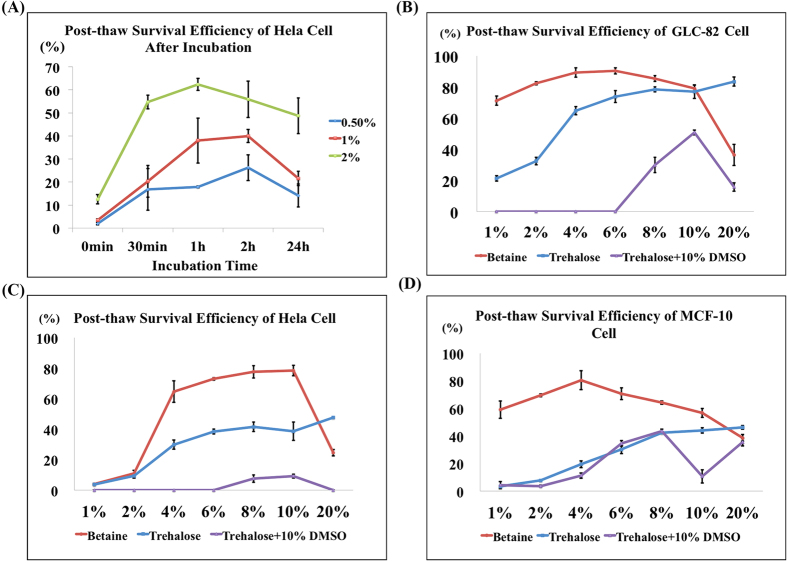
Rapid intracellular bioprotection of betaine. (**A**) Post-thaw efficiency of Hela cells after incubation for different periods in medium containing 0.5% (blue), 1% (red), 2%, (green) of betaine. Post-thaw survival efficiency of (**B**) GLC-82 cells, (**C**) Hela cells and (**D**) MCF-10 cells after cryopreservation using betaine (red), trehalose (blue), and trehalose + 10% DMSO (purple) with ultrarapid freezing protocol. Value = mean ± standard deviation, n ≥ 3.

As mentioned above, the extracellular ice formation will induce the solute injury, while the intracellular ice formation will cause the ice injury. Therefore, the viable cryopreservation is the requirement of optimum CPA concentrations not only outside but also inside the cells. To further explore the exceptional efficiency of betaine as a CPA, we studied the intracellular protection of betaine. Before cryopreservation, Hela cells were incubated in medium containing 0.5%, 1%, and 2% of betaine for different time periods (0.5, 1, 2, and 24 hours). It can be observed in Fig. 5A that the post-thaw survival efficiency of Hela cells could increase with the incubation time, until the optimum efficiency was achieved at 1 or 2 hours. This phenomenon proved that the efficiency of cell cryopreservation could be improved only after betaine entered the cells to prevent intracellular ice formation. Next, in order to confirm that betaine could afford rapid intracellular protection, we compared the post-thaw survival efficiency of betaine and trehalose with ultrarapid freezing protocol (Fig. 5B–D). Trehalose was selected as the control because without pretreatment it was impermeable to cell membrane and could not achieve intracellular bioprotection. As expected, trehalose samples at most concentrations exhibited much lower survival efficiency as compared with betaine samples. It was further proved that betaine could enter the cells in a remarkable rapid manner, because in ultrarapid freezing protocol, the time window allowing cellular entrance of CPA is very limited (few minutes). Although DMSO was reported to increase cell membrane permeability of trehalose in controlled-rate freezing protocol, but Fig. 5B–D showed that it could not work for ultrarapid freezing. These results suggested the unique properties of betaine that could enter the cells instantly to offer intracellular bioprotection and enable cells to survive ultrarapid cryopreservation.

### Proposed mechanism

**Figure 6:**
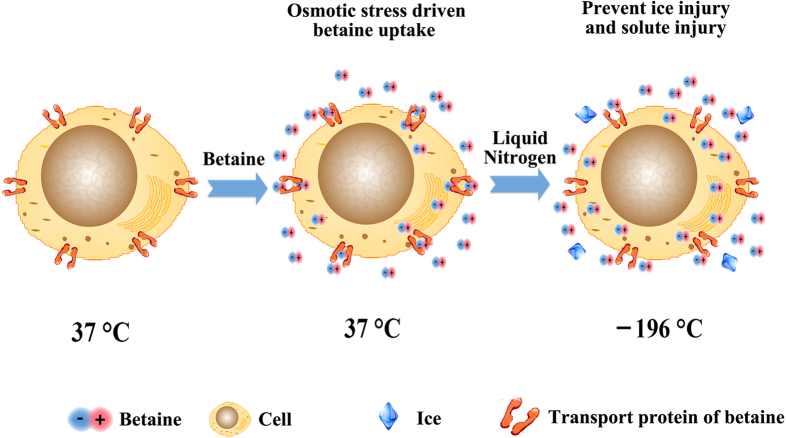
A proposed mechanism of cell cryopreservation using betaine with ultrarapid freezing. During the freezing process, the uptake of betaine by cells via transport proteins is induced by osmotic stress (middle) and prevents the intracellular and extracellular ice injuries as well as solute injury.

From the above results, a potential mechanism can be proposed (Fig. 6). Firstly, betaine was added to form a hypertonic environment for the cells. As an osmoprotectant, betaine could be rapidly taken up by the cells via the transport proteins under hypertonic stress, thus insured the intracellular protection from ice injury^45^. Secondly, ultrarapid freezing was performed to prevent extensive ice growth in contrast to the conventional slow-freezing protocol. In the freezing process, intracellular and extracellular betaine could protect the cells from ice and solute injury, until a glassy state–vitrification–of solution occured (the vitrification temperature of pure water is about −130 °C). Actually, in cryopreservation, cells can only be damaged during the freezing and thawing process; when the aqueous solution vitrifies, further movement of water and ice crystal growth will be halted, and thus the cryo-injuries will also stop^13^. Finally, the undamaged cells will remain intact for long-term storage at −196 °C.

## Conclusions

In summary, we demonstrated that betaine could work as a nontoxic CPA and enable cells to survive ultrarapid cryopreservation to achieve superior cell survival efficiency. It was found that cellular uptake of betaine was ultra-rapid for intracellular protection during the freezing process. With its excellent ability to cryopreserve cells, this natural zwitterionic molecule make it possible to alternate the conventional toxic CPAs for a variety of clinical applications.

## Methods

### Materials

Betaine, trehalose, glucose and NaCl were all purchased from Alfa Aesar. Roswell Park Memorial Institute-1640 (RPMI-1640) and fetal bovine serum (FBS) were all obtained from Gibco. Penicillin-streptomycin (PS), trypsin-EDTA (0.025–0.01%), DMSO, and phosphate buffered saline (PBS) were all purchased from Beijing Solarbio Science and Technology Co. Ltd. The culture medium for GLC-82, Hela, MCF-10 cells contained RPMI-1640 with 10% FBS and 1% PS. Milli-Q water (18.2 MΩ·cm^−1^) was used in all experiments.

### Cell preparation

Hela, GLC-82, MCF-10 cells were all incubated at 37 °C under an atmosphere of 95% air and 5% CO_2_ in RPMI-1640 containing 100 U/mL PS and supplemented with 10% v/v FBS. At confluence, cells were trypsinized with trypsin-EDTA for 5 min to detach the cells from the culture substrates. Cells were then pelleted by centrifugation at 800 rpm for 4 min, resuspended in medium, and finally diluted to the desired concentrations for experiments.

### DSC tests

DSC assessment of ice formation was performed with betaine/water, DMSO/water, and glucose/water mixtures. 40 μL aluminium pans and samples were accurately weighed to ±0.01 mg and transferred to a DSC-1 STAR^e^ System (Mettler-Toledo, DSC 1/500). Heat flow (W/g) was measured and recorded against an empty pre-weighed 40 μL aluminium reference pan starting from −40 °C to 10 °C at 2 °C min^−1^, with the presence of a large endothermic peak demonstrating ice melting. The freezing point depression of water was detected at the beginning of the frozen mixture solutions melt.

### The cell survival efficiency assays

The cell survival efficiency was evaluated using live/dead assays (Live/Dead viability/cytotoxicity kit, Molecular Probes). In the live/dead staining, after calcein-AM/ethidium homodimer-1 reagent mixture solution (100 μL) was introduced to a 96-well TCPS plate containing cells (10 μL), the plates were incubated at room temperature for 30 min away from light, and then observed and analyzed using an inverted microscope (Nikon Eclipse Ti-S). The survival efficiency was calculated by counting the number of live and dead cells in more than 3 different samples.

### Cell-attachment tests

Cells were washed with PBS and suspended in the medium containing 10% FBS. Then, the cell suspension was added into a 24-well TCPS plate. After 12 h, the cell-attachment efficiency and cell morphology were observed using the inverted microscope.

### Osmotic regulation tests

GLC-82 cells were exposed in the medium containing 0.15 M betaine, 0.15 M glucose, 0.15 M NaCl, 0.15 M NaCl + 0.1 M betaine, 0.15 M NaCl + 0.15 M betaine, and 0.15 M NaCl + 0.2 M betaine, respectively. After 1 or 3 days, cell viability was evaluated with live/dead staining. And then, cell-attachment tests were performed as described above.

### Cell cryopreservation with ultrarapid freezing protocol

Different formulations of CPAs (betaine, DMSO, trehalose, or trehalose + DMSO) in medium containing 10% FBS were prepared, and 1.0 × 10^6^ GLC-82, Hela, MCF-10 cells were added to the CPA solutions in cryovials (Corning, 1.8 mL), and the final solution volume was 1.5 mL. Each sample was then directly immersed into liquid nitrogen, and the cooling rate in the cell suspension was about 430 °C/min. After different periods of cryopreservation, cells were immediately thawed at 37 °C (water bath) for evaluating the cell survival efficiency measured as previously described.

### Cytotoxicity test

The cytotoxicity of DMSO and betaine to GLC-82 cells were tested as follows: GLC-82 cells were exposed in the medium containing 2% of DMSO or betaine at 37 °C for 1 to 3 days. After 1, 2 or 3 days, cell attachment efficiency was tested.

### Cell incubation in betaine

1.0 × 10^6^ cells were added to 2 mL medium containing varied concentrations of betaine (0.5%, 1%, and 2%). Then they were added in the culture flask and incubated at 37 °C under an atmosphere of 95% air and 5% CO_2_ for different periods (0.5 h, 1 h, 2 h, 24 h). After that, cells were treated with trypsin-EDTA to detach cells from the culture substrate, and resuspended in the betaine solutions (0.5%, 1%, and 2%), respectively. Then the cells were cryopreserved with ultrarapid freezing protocol. After cryopreservation, cells were immediately thawed at 37 °C (water bath) to evaluate the cell survival efficiency measured as previously described.

## Additional Information

**How to cite this article**: Yang, J. *et al*. Natural zwitterionic betaine enables cells to survive ultrarapid cryopreservation. *Sci. Rep*. **6**, 37458; doi: 10.1038/srep37458 (2016).

**Publisher's note:** Springer Nature remains neutral with regard to jurisdictional claims in published maps and institutional affiliations.

## Supplementary Material

Supplementary Information
