# Supplementary material for: Natural zwitterionic betaine enables cells to survive ultrarapid cryopreservation
Source: Sci Rep. 2016 Nov 22;6:37458. doi: 10.1038/srep37458 (PMC5118695; doi:10.1038/srep37458)
Supplement: Supplementary Information [file srep37458-s1.pdf]

Supplementary Information

Natural zwitterionic betaine enables cells to survive ultrarapid cryopreservation

Jing Yang[1,2,3], *Nana Cai*[1,2,3], *Hongwen Zhai*[1,2,3], *Jiamin Zhang*[1,2,3], *Yingnan Zhu*[1,2,3], *Lei Zhang*[1,2,3,]*

[1] Department of Biochemical Engineering, School of Chemical Engineering and Technology, Tianjin University, Tianjin 300072, PR China

[2] Key Laboratory of Systems Bioengineering of the Ministry of Education, Tianjin University, Tianjin 300072, PR China

[3] Collaborative Innovation Center of Chemical Science and Engineering (Tianjin), Tianjin University, Tianjin, 300072, PR China

E-mail: lei_zhang@tju.edu.cn

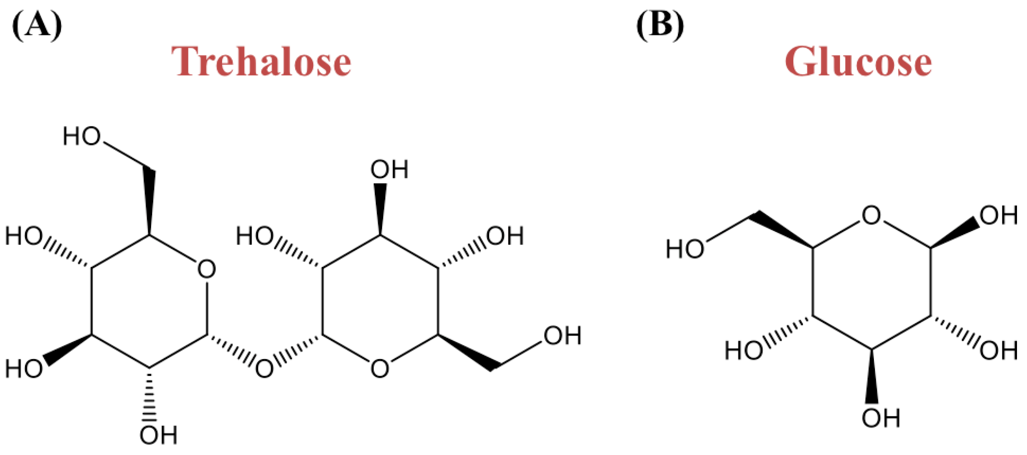

Fig. S1. The molecular structure of (A) trehalose and (B) glucose.

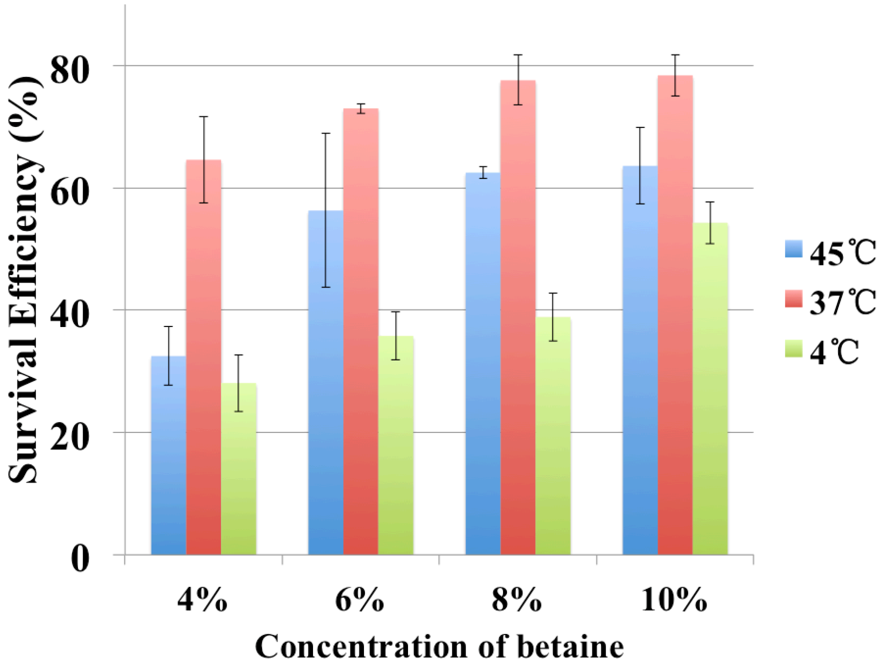

Fig. S2. Effect of thawing temperature on survival efficiency of Hela cell cryopreservation. Post-thaw survival efficiency of Hela cells cryopreserved with 4%, 6%, 8%, 10% of betaine at thawing temperature 45 °C (blue), 37 °C (red), 4 °C (green). Value= mean ± standard deviation, n≥3.

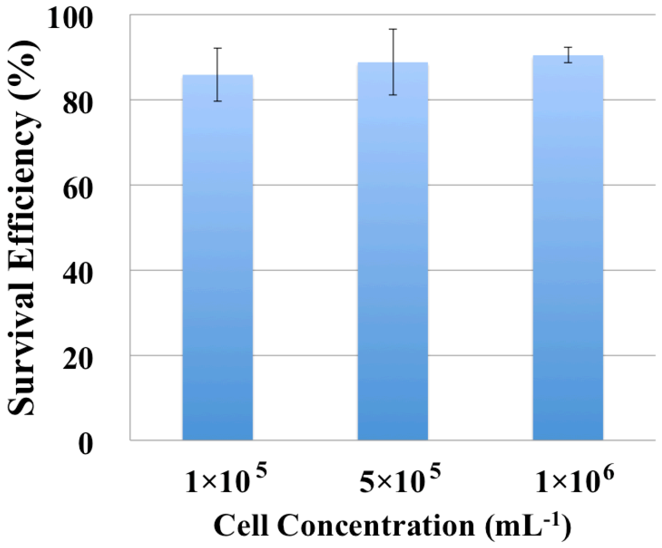

Fig. S3. Post-thaw survival efficiency of GLC-82 cells with different cell concentrations. Value= mean ± standard deviation, n≥3.

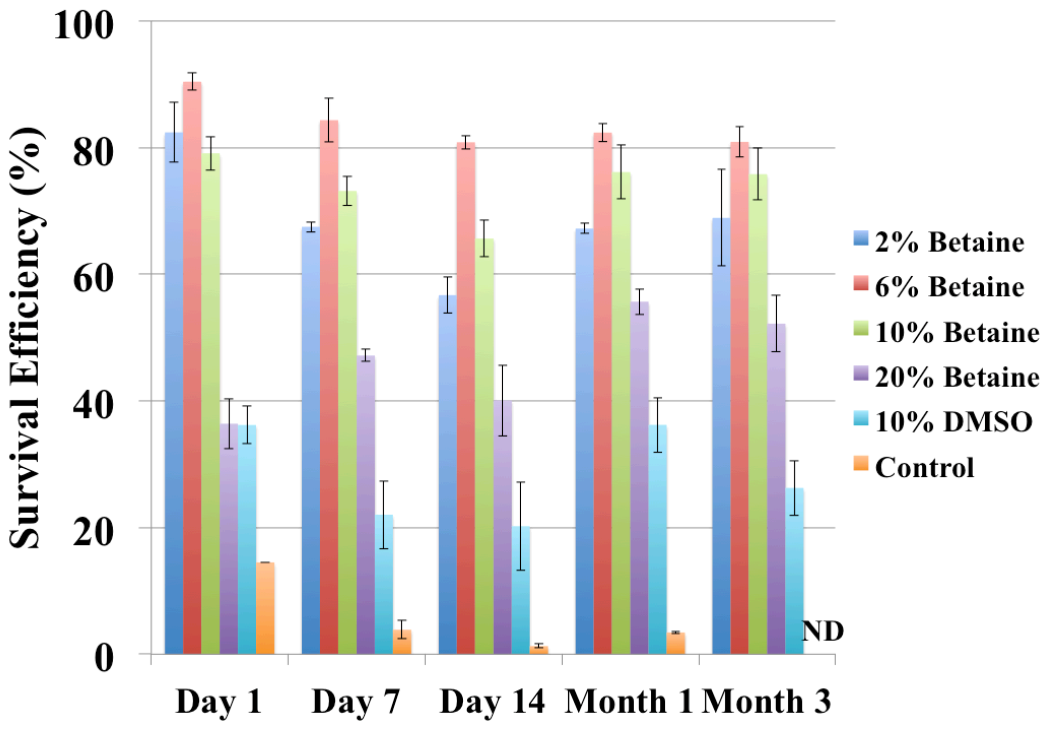

Fig. S4. Post-thaw survival efficiency of GLC-82 cell cryopreservation for different periods from 1 day to 3 month with 2% (dark blue), 6% (red), 10% (green), 20% (purple) of betaine, 10% of DMSO (light blue), and culture medium as a control (orange). Value= mean ± standard deviation, n≥3.

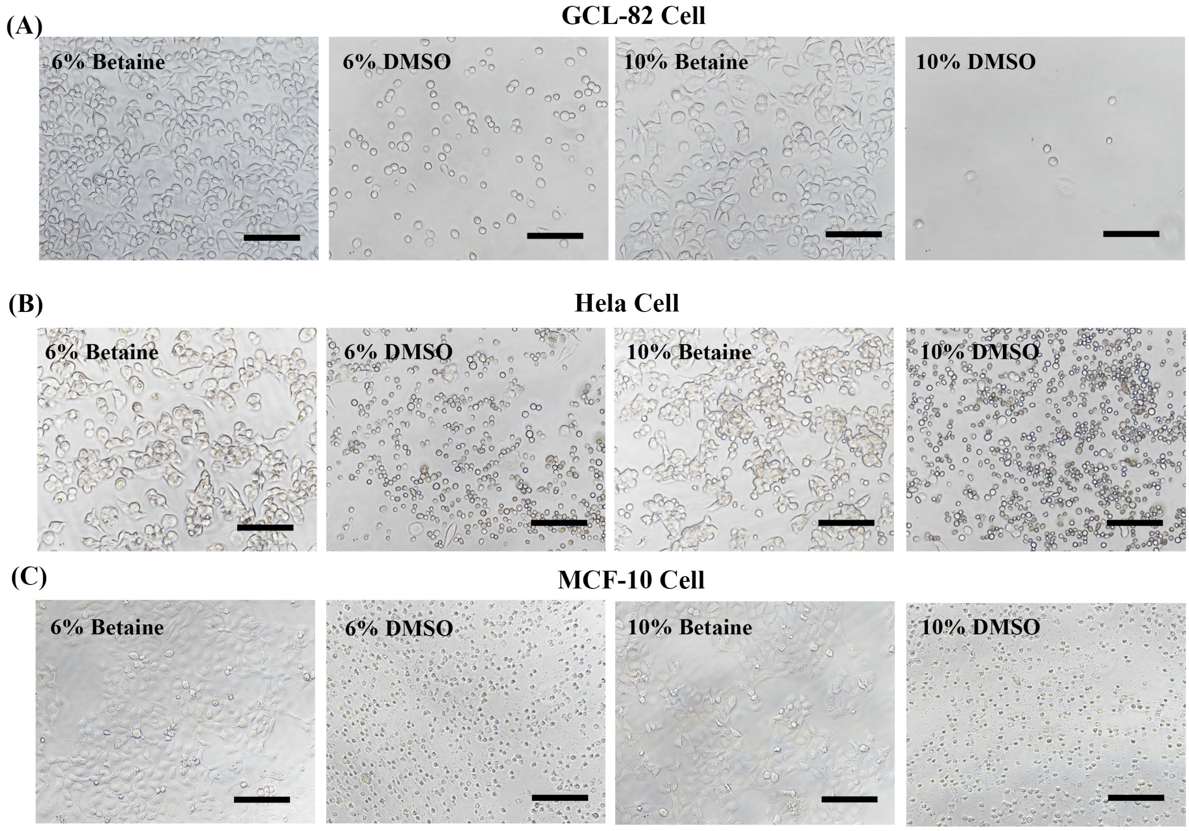

Fig. S5. Post-thaw recovery of (A) GLC-82, (B) Hela, and (C) MCF-10 cells. Three cell types were cryopreserved using 6% or 10% of CPAs (betaine or DMSO), respectively, and then the post-thaw cells with CPAs solution added four-fold amount of fresh medium were directly infused into a culture flask for cell recovery with the wash steps skipped. Scale bar = 50 μm.

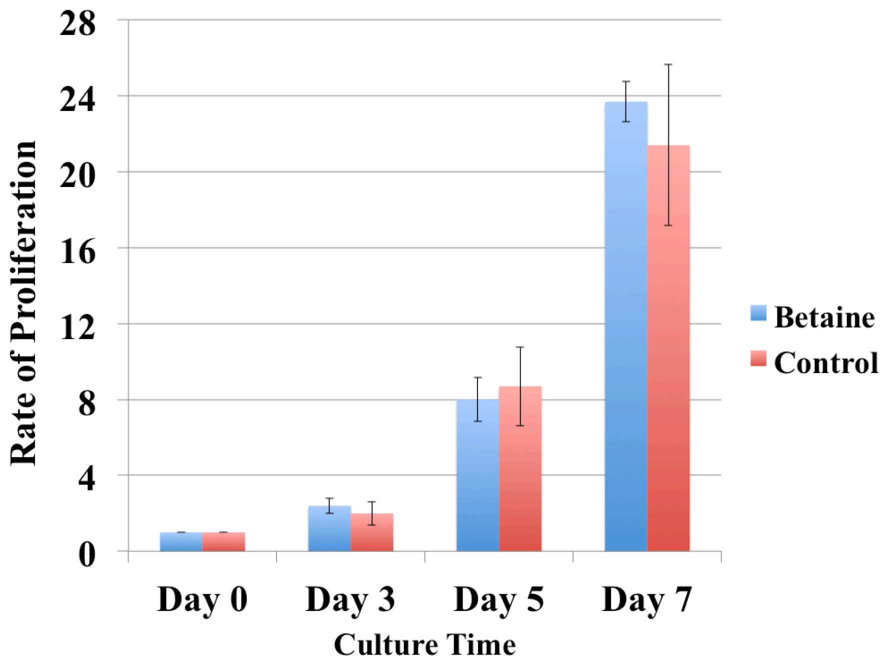

Fig. S6. Comparison of the proliferation rate (total cell numbers/ initial cell numbers) of GLC-82 cells in a culture flask after cryopreservation using betaine versus the cells without cryopreservation. Value= mean ± standard deviation, n≥3.
